# Enhancing chemotherapeutic efficacy: Niosome‐encapsulated Dox‐Cis with MUC‐1 aptamer

**DOI:** 10.1002/cam4.70079

**Published:** 2024-08-09

**Authors:** Firat Baris Barlas, Bilge Olceroglu, Didem Ag Seleci, Zinar Pinar Gumus, Pinar Siyah, Meriam Dabbek, Georg Garnweitne, Frank Stahl, Thomas Scheper, Suna Timur

**Affiliations:** ^1^ Institute for Technical Chemistry Leibniz University Hannover Hannover Germany; ^2^ Institue of Nanotechnology and Biotechnology İstanbul University‐Cerrahpaşa İstanbul Turkey; ^3^ Institute for Particle Technology (iPAT) Technische Universität Braunschweig Braunschweig Germany; ^4^ Central Research Test and Analysis Laboratory Application and Research Center Ege University Izmir Turkey; ^5^ Department of Biochemistry, School of Pharmacy Bahçeşehir University Istanbul Turkey; ^6^ Department of Biochemistry, Faculty of Science Ege University Izmir Turkey

**Keywords:** cisplatin, combine drug, doxorubicin, MUC‐1, niosome

## Abstract

**Background:**

Cancer remains a formidable global health challenge, currently affecting nearly 20 million individuals worldwide. Due to the absence of universally effective treatments, ongoing research explores diverse strategies to combat this disease. Recent efforts have concentrated on developing combined drug regimens and targeted therapeutic approaches.

**Objective:**

This study aimed to investigate the anticancer efficacy of a conjugated drug system, consisting of doxorubicin and cisplatin (Dox‐Cis), encapsulated within niosomes and modified with MUC‐1 aptamers to enhance biocompatibility and target specific cancer cells.

**Methods:**

The chemical structure of the Dox‐Cis conjugate was characterized using Fourier Transform Infrared Spectroscopy (FTIR) and Liquid Chromatography Quadrupole Time‐of‐Flight Mass Spectrometry (LC‐Q‐TOF/MS). The zeta potential and morphological parameters of the niosomal vesicles were determined through Dynamic Light Scattering (DLS) and Transmission Electron Microscopy (TEM). In vitro assessments of cell viability and apoptosis were conducted on MUC‐1 positive HeLa cells and MUC‐1 negative U87 cells.

**Results:**

The findings confirmed the successful conjugation of Dox and Cis within the niosomes. The Nio/Dox‐Cis/MUC‐1 formulation demonstrated enhanced efficacy compared to the individual drugs and their unencapsulated combination in both cell lines. Notably, the Nio/Dox‐Cis/MUC‐1 formulation exhibited greater effectiveness on HeLa cells (38.503 ± 1.407) than on U87 cells (46.653 ± 1.297).

**Conclusion:**

The study underscores the potential of the Dox‐Cis conjugate as a promising strategy for cancer treatment, particularly through platforms that facilitate targeted drug delivery to cancer cells. This targeted approach could lead to more effective and personalized cancer therapies.

## Graphical abstract

Schematic illustration of the conjugation of doxorubicin and cisplatin (Dox‐Cis), followed by encapsulation within niosomes and functionalization with MUC‐1 aptamers for targeted cancer therapy. (Created with BioRender.com)

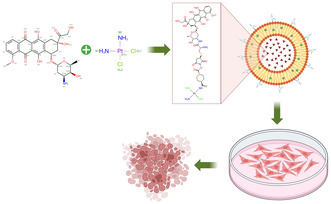

## 1 INTRODUCTION

Globally, cancer is the second leading cause of death after cardiovascular diseases. In 2024 alone, it is projected to cause 2,001,140 new cancer cases and 611,720 cancer deaths in the United States.^1^ Cancer, characterized by the abnormal and uncontrolled growth of cells leading to tumor formation, spreading to other tissues, damaging them, and creating its own microenvironment, remains highly fatal with no definitive cure. Despite its numerous types and varied causes of cancer, one of the most commonly employed treatments today is chemotherapy. However, the significant side effects of chemotherapy, drug resistance, and the inability to implement personalized treatment methods pose substantial limitations in this process.^2^ Recent advances in nanotechnology have shown the potential to revolutionize cancer treatment.^3^, ^4^ The encapsulation of chemotherapeutic agents within nanocarriers, such as niosomes, enhances their circulation time in blood, facilitates the co‐administration of multiple drugs, and supports targeted therapy.^3^, ^5^ Niosomes, which are synthetic vesicles constructed from non‐ionic surfactants and cholesterol,^3^ are particularly valued for their ability to encapsulate diverse pharmacological agents, accommodating hydrophilic, amphiphilic, and lipophilic molecules. These vesicles may also be functionalized with active end groups like polyethylene glycol (PEG) to enhance their pharmaceutical attributes. The advantages of niosomes, including their cost‐effectiveness, chemical stability, biocompatibility, and biodegradability, make them attractive for controlled drug release and targeted therapy.^6^, ^7^, ^8^ Furthermore, their unique structures allow for the simultaneous use of multiple strategies such as drugs, hydrogels, nanoparticles, and polymers. In addition, they possess the capacity to carry both water‐soluble and water‐insoluble agents simultaneously.^9^

Aptamers, low molecular weight single‐stranded DNA or RNA molecules (6–30 kd),^10^ adopt specific tertiary structures enabling them to bind selectively to molecular targets.^11^ Due to their stability under non‐physiological conditions, biocompatibility, and high penetration capabilities, aptamers are increasingly utilized as a cost‐effective alternative to antibodies in targeted therapy.^11^ Mucin‐1 (MUC‐1), a macromolecular transmembrane protein, forms a dense network on epithelial cell surfaces, providing protection against environmental threats, and playing a significant role in oncogenesis.^12^, ^13^, ^14^ MUC‐1 has been reported to be overexpressed in various cancer tissues.^15^ Abnormal expression of MUC‐1 causes a decrease in the polarity of epithelial cells and alters the signaling directions in the area where it acts.^15^ For this reason, MUC‐1 has been the focus of attention by researchers in cancer treatment.^16^

The limitations of traditional chemotherapy, such as adverse side effects and cellular resistance, have necessitated the exploration of alternative therapeutic strategies,^17^ with combination therapies often employed to enhance treatment efficacy.^18^ Doxorubicin (Dox), an anthracycline anticancer agent^19^ and a topoisomerase II inhibitor, interferes with DNA replication by binding to the DNA.^20^ Topoisomerase II enzyme is involved in chromosome condensation, separation of intertwined DNA strands, and relaxation of tension in DNA strands during replication.^19^ Cisplatin (Cis), another well‐established chemotherapy drug, forms platinum‐DNA adducts with purine bases, disrupting DNA transcription and replication processes.^21^ Cis has a cytotoxic effect by binding to the N7 region of the guanine base.^22^ The chloride ligands allow the platinum ion to make interactions with DNA bases, whereas the ammonia ligands in the Cis structure bind more strongly with the protein.^23^ Chlorine atoms are replaced by hydrolyzed and activated water molecules when Cis enters the cell.^23^

**FIGURE 1:**
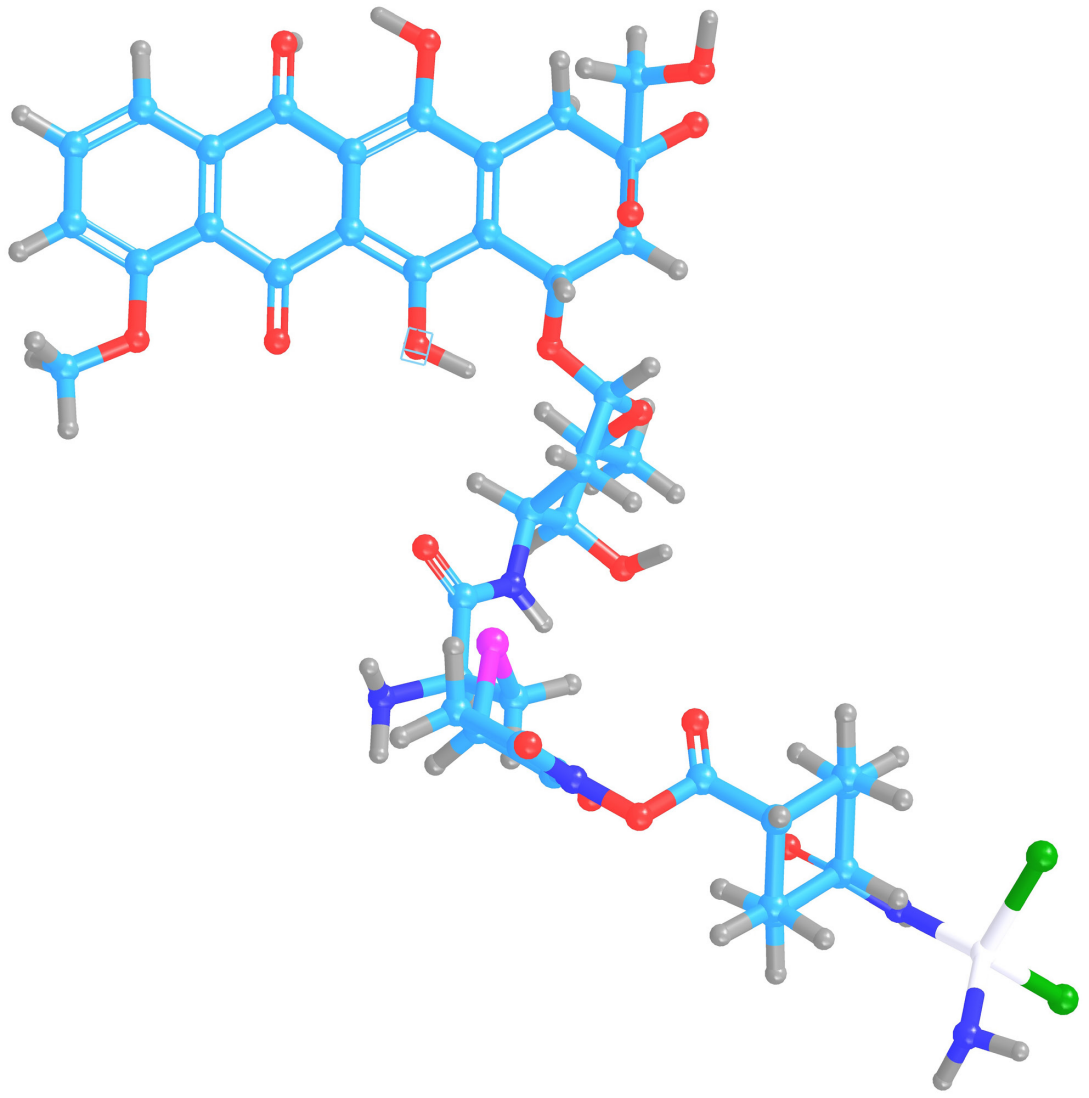
3D structure of Dox‐Cys‐Cis conjugate. The compound was designed using a 2D Sketcher module of Maestro program (1194.919 MW). Energy minimization was performed using AutoDock Vina program, and the resulting structure was visualized in the Chimera X program.

Studies conducted with DOX‐Cis conjugated drugs increased the chemotherapeutic potential of both drugs.^24^, ^25^ However, DOX‐Cis conjugated drug trials reduced cytotoxicity and provided tumor inhibition.^26^ This study explores the synergistic potential of a conjugated system comprising doxorubicin and cisplatin, with cysteine employed to facilitate their conjugation (Figure 1). The hypothesis is that the distinct mechanisms of Dox and Cis could be leveraged to enhance the cytotoxic effects when combined.^27^ The resulting conjugates were encapsulated in targeted niosomes. Subsequent characterization experiments assessed the impact of Nio/Dox‐Cis/MUC‐1 on HeLa and U87 cell lines, revealing pronounced cytotoxic effects. This suggests that conjugation and targeted delivery might amplify the therapeutic efficacy of the drug combination.

## 2 EXPERIMENTAL SECTION

### 2.1 Materials

DSPE‐PEG (2000) Amine and cholesterol were sourced from Avanti Polar, while the SH‐MUC‐1 aptamer S2.2 was acquired from Integrated DNA Technologies. Additional chemicals including cisplatin, doxorubicin, 1‐ethyl‐3‐(3‐dimethylaminopropyl) carbodiimide hydrochloride (EDC), phosphate‐buffered saline (PBS, pH 7.4), MES buffer (pH 6.0), FITC annexin V, N‐hydroxysuccinimide (NHS), sulfo‐SMCC, Tween 61, and CellTiter‐Blue (CTB) were procured from Sigma‐Aldrich. Chloroform, methanol, and 2‐propanol (all HPLC grade) were also obtained from Sigma‐Aldrich, while ultrapure water was produced using a Milli‐Q plus System (Millipore). The masses of the synthesized materials were determined using an Agilent 6550 iFunnel High‐Resolution Accurate‐Mass Q‐TOF/MS equipped with an Agilent Dual Jet Stream electrospray ionization (Dual AJS ESI) interface in positive ion mode.

### 2.2 Conjugation of doxorubicin to cysteine

Doxorubicin and cisplatin were conjugated using the EDC‐NHS protocol as delineated in the methodology proposed by Geyik et al.^28^ The conjugation procedure commenced by combining 0.1 M cysteine with 0.2 M 1‐ethyl‐3‐(3‐dimethylaminopropyl) carbodiimide hydrochloride (EDC) and 0.05 M N‐hydroxysuccinimide (NHS) in a 500 μL MES buffer solution at pH 6.0 (25 mM concentration). This mixture was placed within a thermo shaker set at room temperature and agitated at 1000 rpm for 15 min, a step which facilitated the activation of the carboxyl groups on cysteine. Following this activation, 0.1 M doxorubicin was introduced to the activated mixture and allowed to incubate for an additional 2 h, during which the conjugation between doxorubicin and the activated cysteine molecules occurred. Post‐conjugation, the mixture was subjected to overnight dialysis using a membrane with a molecular weight cutoff of 0.1–0.5 kDa in PBS at pH 7.4 to remove any unbound molecules. The conjugates isolated post‐dialysis were then used for further experimental procedures.

### 2.3 Conjugation of doxorubicin–cysteine to cisplatin

The conjugation of doxorubicin‐cysteine with cisplatin was performed using the sulfo‐SMCC methodology as described by Liu et al.^29^ Initially, a solution of 0.5 M cisplatin was prepared and combined with 0.1 M sulfo‐SMCC in PBS. This mixture was then augmented by the addition of the doxorubicin–cysteine (Dox‐Cys) conjugate, which was allowed to react for 30 min at ambient temperature. Following this initial reaction phase, the mixture was subjected to an additional 30 min of incubation in a thermo shaker maintained at room temperature to facilitate the conjugation between cisplatin and the Dox‐Cys conjugate. Post‐incubation, the reaction mixture underwent overnight dialysis using a membrane with a molecular weight cutoff of 0.5 kDa in PBS (pH 7.4), aimed at removing unbound molecules and any excess reagents or unreacted components. The resultant Dox‐Cis conjugates, isolated following dialysis, were then employed in subsequent experimental investigations.

### 2.4 Synthesis of niosomes

Ag Seleci and colleagues formulated PEGylated niosomes via the film hydration method, detailed in their publication.^30^ Initially, a mixture consisting of Tween 61, cholesterol, and DSPE‐PEG (2000) Amine was prepared at a molar ratio of 1:1:0.1 and dissolved in 1.0 mL of chloroform within a round‐bottom flask. The chloroform solvent was subsequently removed under reduced pressure using a rotary evaporator. Following solvent evaporation, the resultant thin lipid film was rehydrated with 1.0 mL of an aqueous solution containing the Dox‐Cis conjugate at 60°C for a duration of 60 min. This process facilitated the formation of small unilamellar vesicles (SUVs), which were then extruded through mini extruders equipped with polycarbonate filters of 0.4 and 0.1 mm pore sizes, supplied by Avanti Polar Lipids. The volume of the lipid suspension used for extrusion was maintained at 1.0 mL.

### 2.5 Conjugation of aptamer

The conjugation of amine‐functionalized niosomes with thiol‐modified (‐SH) aptamers was conducted utilizing the sulfo‐SMCC coupling protocol. Initially, the niosomes were incubated with sulfo‐SMCC in PBS at ambient temperature for 30 min. Following this incubation, the aptamers were introduced into the reaction mixture. The combined solution, containing both niosomes and aptamers, was subsequently agitated in a thermo shaker for an additional 30 min at room temperature to facilitate the conjugation process. Post‐conjugation, the reaction mixture was subjected to dialysis using an appropriate dialysis membrane to remove unbound peptides and excess aptamers. This dialysis step was crucial in ensuring the isolation of the desired niosome–aptamer complexes, free from any unbound or excess components.

### 2.6 Encapsulation efficiency

The encapsulation efficiency (%EE) of the doxorubicin–cisplatin (Dox‐Cis) loaded niosomes was quantified based on the fluorometric properties of doxorubicin. To conduct this analysis, niosome samples that had been freshly dialyzed were utilized. The vesicle bilayer was disrupted by vortexing and subsequent sonication in methanol for 10 min. A calibration curve for doxorubicin was established using standard solutions of doxorubicin prepared in PBS at concentrations ranging from 1.0 to 50 μM. The disrupted Dox‐loaded niosomes were then diluted with PBS, and the concentration of encapsulated doxorubicin was determined by comparing the fluorescence intensity to the established calibration curve.

### 2.7 Particle size

The particle size and zeta potential of Nio/Dox‐Cis/MUC‐1 were assessed using dynamic light scattering (DLS) analysis, conducted with a Malvern Zetasizer Nanoseries‐Nano‐ZS. Additionally, the size and morphology of the particles were examined through transmission electron microscopy (TEM) using a JEM‐2100F microscope (JEOL, Tokyo, Japan).

### 2.8 Fourier transform infrared

The conjugation of Dox‐Cis following lyophilization was analyzed by obtaining FTIR spectra, specifically using the attenuated total reflection (ATR) technique. The ATR‐FTIR measurements were performed with a spectrometer from Bruker, located in Billerica, MA, USA.

### 2.9 Chromatographic analysis

The molecular mass of the synthesized compound was confirmed through liquid chromatography quadrupole time‐of‐flight mass spectrometry (LC‐Q‐TOF/MS) analysis. Prior to analysis, the sample solution was filtered using a 0.22 μm filter to ensure purity before introduction into the LC‐Q‐TOF/MS system. The chromatographic separation was conducted on an HPLC Agilent 1260 Infinity series system, which is equipped with a binary pump, online degasser, autosampler, and a Poroshell 120 EC‐C18 column (3.0 × 50 mm, 2.7 μm particle size) from Agilent Technologies. The mobile phase comprised 0.1% formic acid in water (solvent A) and acetonitrile (solvent B). A gradient elution protocol was implemented starting with 10% B for the initial 0.5 min, escalating to 70% B over the next 4.5 min, increasing to 95% B from 5 to 7 min, holding at 95% B until 10 min, and then returning to 10% B from 10 to 15 min to re‐equilibrate the column. The column temperature was consistently maintained at 35°C. For the analysis, 2.0 μL of the sample was injected, and the flow rate was set at 0.5 mL/min, facilitating efficient component separation and precise mass determination.

Mass spectrometry (MS) analysis was performed using an Agilent 6550 iFunnel high‐resolution Accurate‐Mass QTOF‐MS system, which is equipped with an Agilent Dual Jet Stream electrospray ionization (Dual AJS ESI) interface and was operated in positive ion mode. The specific parameters set for the analysis included a drying gas flow rate of 14.0 L/min, nebulizer pressure at 35 psi, gas drying temperature at 290°C, sheath gas temperature at 400°C, and a sheath gas flow rate of 12 L/min. The mass scanning range was established from m/z 50 to 2000, with a collision energy of 10 eV applied during the analysis. Data acquisition and subsequent processing were conducted using the MassHunter Workstation software, developed by Agilent Technologies, based in Santa Clara, CA, USA. This software facilitated the integration and evaluation of the MS data, ensuring the accuracy and reliability of the results.

### 2.10 Cell culture

The U87 and HeLa cell lines were obtained from the German Collection of Microorganisms and Cell Cultures (DSMZ). These cell lines were cultured in Dulbecco's modified Eagle medium (DMEM) enriched with 10% Fetal Calf Serum (FCS) and 1.0% penicillin/streptomycin (P/S). The culturing was conducted at 37°C in a humidified incubator with an atmosphere of 5.0% CO_2_. Subsequent to the culturing phase, the cells underwent various treatments with designated samples and reagents as required for the experimental procedures.

### 2.11 Cytotoxicity

Cell viability was evaluated utilizing the CellTiter‐Blue Assay, adhering to the protocol delineated by Kovalchuk.^31^ Initially, 4 × 10^3^ cells were distributed across each well of a 96‐well microplate. Upon achieving 70%–80% confluence, the cells were treated with CellTiter‐Blue reagent (CTB) and incubated for a duration of 2 h. Following incubation, fluorescence intensities were quantified using a Fluoroskan Ascent fluorescence spectrometer (Thermo Fisher Scientific Inc., Waltham, MA, USA). Measurements were conducted at an excitation wavelength of 544 nm and an emission wavelength of 590 nm.

### 2.12 Cellular uptake and internalization

To evaluate the cellular uptake of Nio/Dox‐Cis/MUC‐1, the Cytation 5 imaging reader from Biotek, USA, was employed. Initially, 8 × 10^3^ cells were plated in 96‐well plates and cultured for 48 h under standard conditions. Following this incubation period, the culture medium was removed, and the cells were exposed to non‐toxic concentrations of the Nio/Dox‐Cis/MUC‐1 formulation in the treatment medium. After a further 2‐h incubation, the cells were washed twice with PBS.^32^ Imaging was subsequently performed using the Cytation 5 imaging reader, capturing both bright field and fluorescence images under red filter conditions.

### 2.13 Statistical analysis

All experiments in this study were conducted with a minimum of three replicates, and the data are reported as mean ± standard deviation (SD), except where otherwise indicated. Statistical evaluations were carried out using a two‐way analysis of variance (ANOVA), followed by Tukey's multiple comparison test to assess the significance of differences between experimental groups (GraphPad Prism 9). A significance threshold of *p* < 0.05 was established for determining statistically significant differences. *p* < 0.001 or 0.0001 were considered to indicate highly significant differences.

## 3 RESULTS AND DISCUSSION

### 3.1 Conjugation and characterization of Dox‐Cis

In contemporary oncology, the concurrent administration of multiple drugs is frequently favored for cancer treatment. Although the use of combination drug therapy, particularly when involving chemotherapeutic agents, can introduce toxicity, this risk is often mitigated by the targeting of distinct biochemical pathways. Such an approach can result in synergistic or additive therapeutic effects, thereby allowing for the administration of lower dosages of each drug, reducing overall toxicity.^33^ Doxorubicin and cisplatin, two commonly utilized chemotherapeutic agents, are effective in treating various cancers but are associated with significant side effects. Moreover, cancer cells often develop resistance, surviving despite the presence of these therapeutic agents.^27^, ^34^

In this study, the synergistic interaction between doxorubicin and cisplatin was explored following their conjugation. Cysteine served as an intermediary molecule in the conjugation process between doxorubicin and cisplatin. Initially, doxorubicin (Dox) and cysteine (Cys) were conjugated through EDC/NHS chemistry targeting the NH2 and COOH functional groups, resulting in a Dox‐Cys conjugate with a terminal ‐SH group. Following this, cisplatin, which possesses an NH_2_ group, was attached to the ‐SH group of the Dox‐Cys conjugate using the sulfo‐SMCC method. Excess and unreacted molecules were subsequently removed through dialysis. The resultant conjugates were characterized using FTIR spectroscopy, liquid chromatography quadrupole time‐of‐flight mass spectrometry (LC‐Q‐TOF/MS), and nanodrop fluorimetry techniques.

**FIGURE 2:**
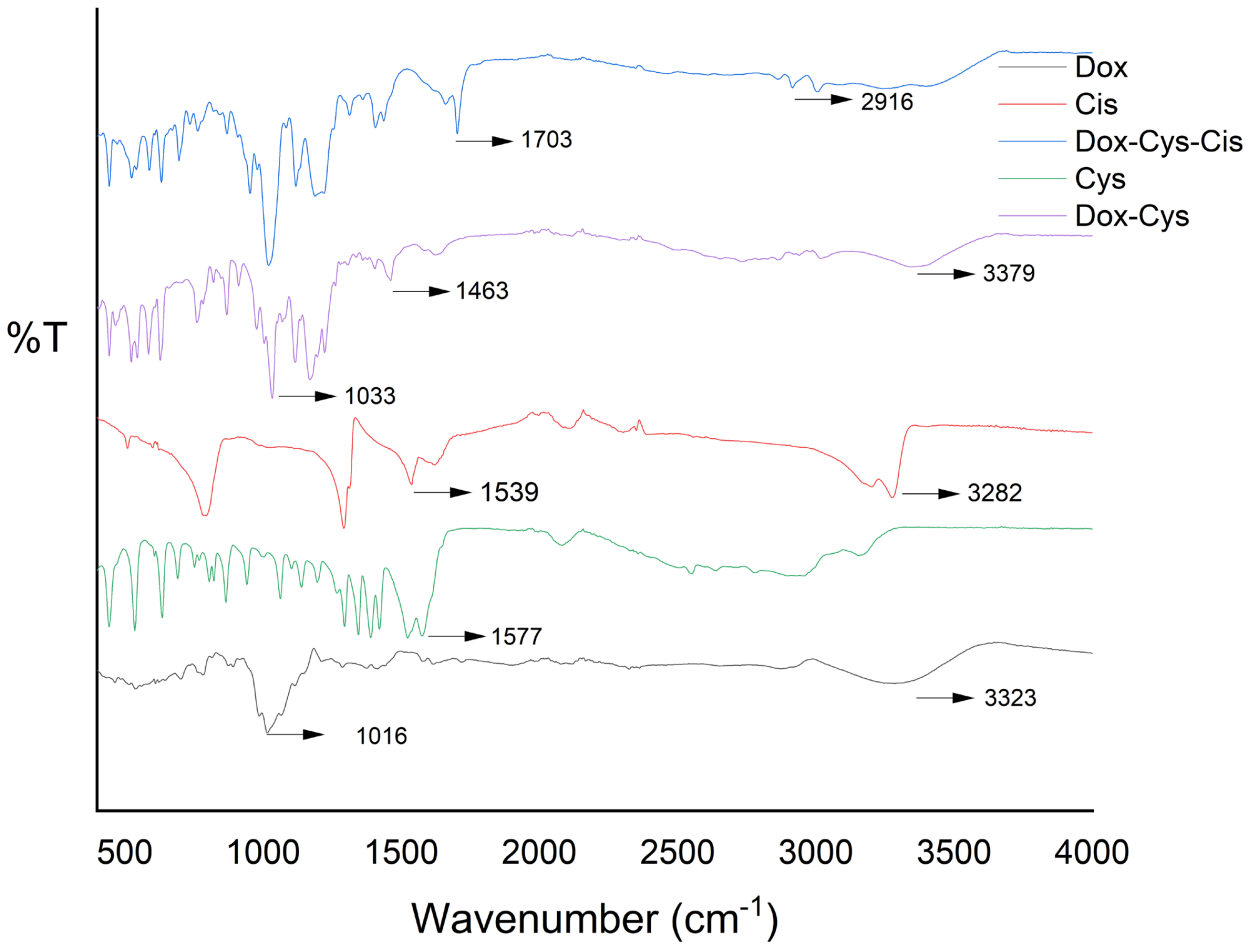
FTIR spectra of free dox, free Cys, free Cis, Dox‐Cys conjugates, and Dox‐Cys‐Cis conjugate.

To elucidate the differences between samples in FTIR spectroscopy, the study was structured into two distinct parts. In the initial segment, FTIR spectroscopy was utilized to analyze free cysteine, free doxorubicin, and the Dox‐Cys conjugate. The spectroscopic results revealed distinct peaks at 1577, 1033, 1463, 3379, and 1016 cm^−1^. The peak at 1577 cm^−1^ is attributed to the COO‐ stretch of the cysteine molecule.^35^ The peaks at 1033 and 1016 cm^−1^ correspond to the C‐O‐C group,^36^ while 1463 cm^−1^ is indicative of the amide bond.^37^ The peak at 3379 cm^−1^ represents the ‐OH band.^38^ Notably, the absorption bands at 1033, 1463, and 3379 cm^−1^ confirm the presence of both Cys and Dox within the conjugate. In the second part of the study, FTIR spectra were obtained for Dox‐Cys and Dox‐Cys‐Cis conjugates in comparison with free cisplatin. The spectroscopy results displayed distinct peaks at 1703, 2916, 1539, and 3282 cm^−1^ for the Dox‐Cys‐Cis and free cisplatin samples (Figure 2). The first two peaks correlate with the benzenethiol group and the sulfhydryl group, respectively,^39^ while the latter two peaks are characteristic of cisplatin, representing ‐NH boundaries. The presence of these characteristic bands provides pivotal evidence for the successful conjugation of the components.

**FIGURE 3:**
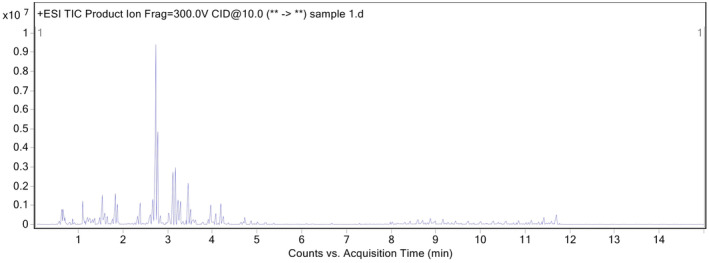
Total ion chromatogram of synthesized compound.

**FIGURE 4:**
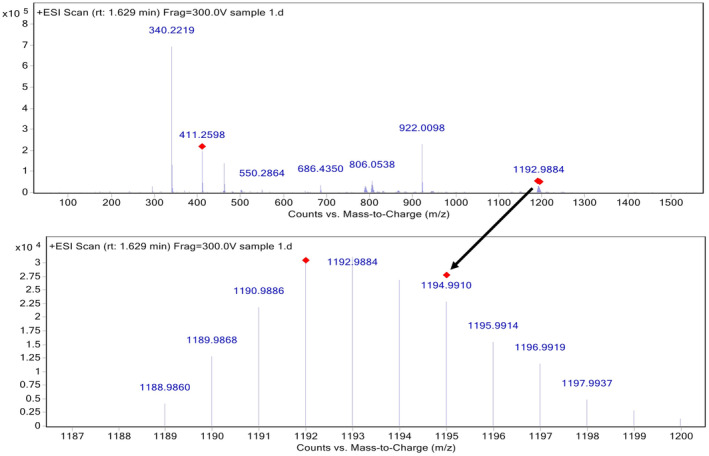
Mass spectrum of 1194.2026 *m*/*z* for 1.629 min.

The possible calculated exact mass of the synthesized compound is 1193.1947 *m*/*z*. Figure 3 shows the total ion chromatogram (TIC) of the conjugate. The chromatographic peak with a retention time of 1.63 min was analyzed and found to contain a significant quantity of mass. The mass spectrometric data corresponding to a M + 1 (1194.2026) ion is presented, including detailed mass spectra of this specific mass region, as depicted in Figure 4. These results are consistent with the molecular weight calculations performed using the Canvas module of the Maestro molecular modeling package.

When the theoretical mass of the synthesized compound and the experimentally obtained masses are examined, it is seen in the mass spectrum that the experimental masses are closely compatible with the theoretical mass predictions of the synthesized compound.

**FIGURE 5:**
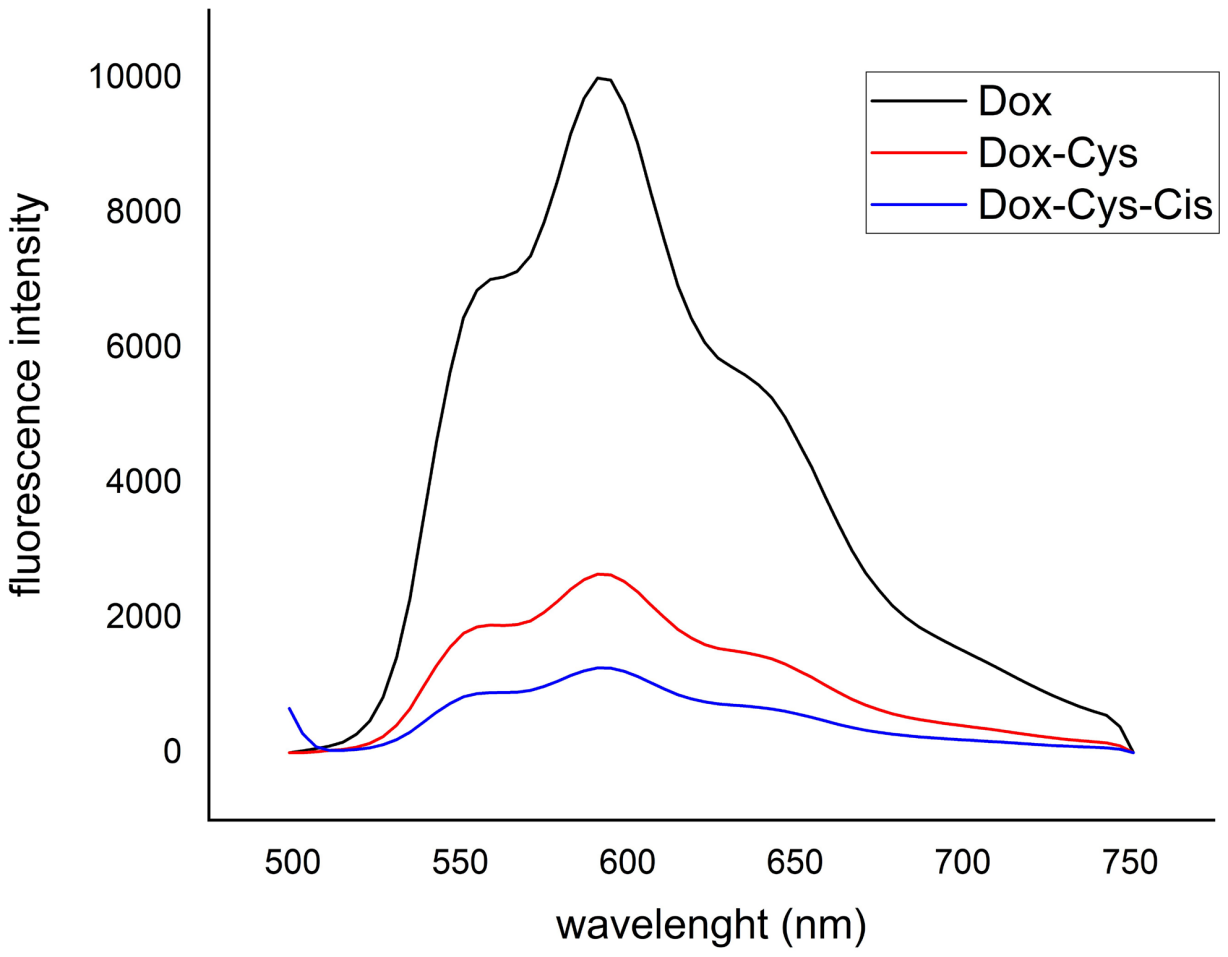
Fluorescence spectrum of free Dox, Dox‐Cys, and Dox‐Cys‐Cis.

Fluorometric analysis of the conjugate was conducted using a Nanodrop device (Thermo Scientific). Specifically, fluorescence measurements for free doxorubicin (Dox), Dox‐Cys, and Dox‐Cys‐Cis were recorded at approximately 590 nm. The fluorescence spectra, depicted in Figure 5, illustrate slight shifts and reductions in fluorescence intensity subsequent to the conjugation processes, indicating alterations in the chemical environment of the doxorubicin molecule post‐conjugation.

**FIGURE 6:**
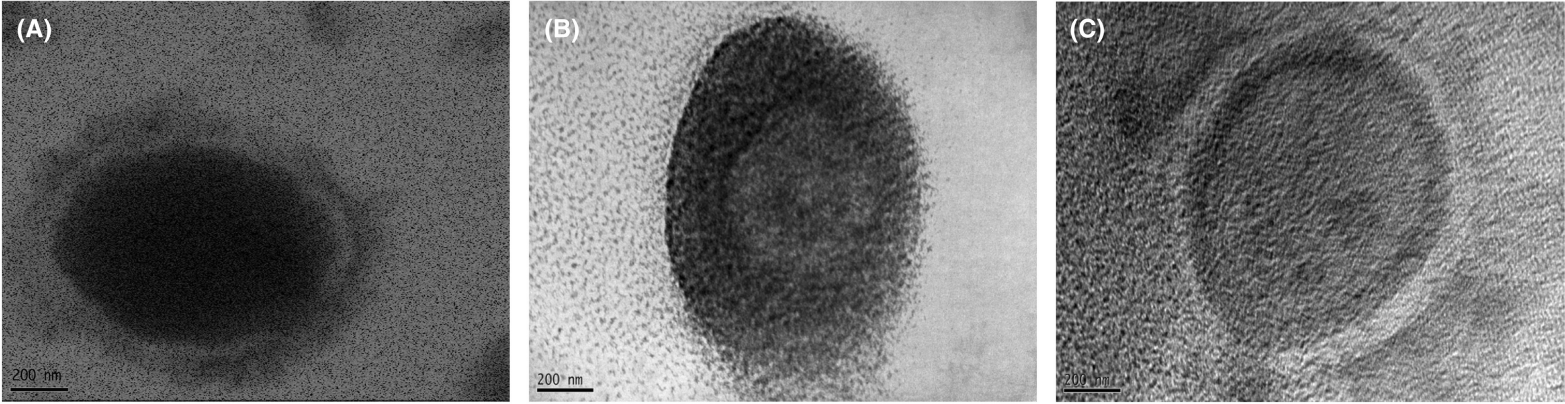
TEM images of vesicular cargos (A) empty noisome (control group) (B) and (C) Nio/Dox‐Cis/MUC1 (scale bar 200 nm).

To enhance the biocompatibility and extend the circulation time of the conjugates in the bloodstream, PEGylated niosomes were synthesized. These niosomes were sized to the desired specifications using a mini extruder, and active targeting was achieved through the conjugation of MUC‐1 aptamers. The particle size and zeta potential of these niosomes were evaluated using DLS analysis, while their size and morphology were further assessed via TEM. For drug delivery systems, the expected vesicle size is between 50 and 200 nm. Larger structures have the potential to be easily eliminated within living tissues. It has been indicated that they can be recognized by immunological pathways.^40^ Moreover, it has been demonstrated that as the vesicle size decreases, the efficiency of drug delivery to intercellular spaces and cells increases.^41^ The resulting average diameters, polydispersity index (PDI), and zeta potential values are presented in Table 1. It was observed that the sizes of the niosomes decreased following MUC‐1 binding, and the PDI values indicated homogeneity in vesicle size. The significance of size in drug transport has been corroborated by previous studies which reported similar niosome sizes yielding positive outcomes.^42^ Additionally, TEM imaging suggested that the niosomes appeared somewhat larger than indicated by DLS measurements (Figure 6), likely due to swelling of the dried niosomes on the slide.

**TABLE 1:** Size distribution and zeta potential of plain noisome and Nio/Dox‐Cis/MUC1.

	Particle size (nm)	Zeta potential (mV)	Polydispersity index (PDI)
Nio	129 ± 50.38	−1.75 ± 6.45	0.171
Nio/Dox‐Cis/MUC‐1	160 ± 36.25	−21 ± 1.75	0.205

Subsequent experiments confirmed the homogeneity and appropriate sizing of the vesicles. The fluorometric method was utilized to determine the encapsulation efficiency (%EE) of the vesicles, marking the next phase in the characterization process. Given the strong fluorescence of doxorubicin at 590 nm,^43^ encapsulation efficiency was calculated using doxorubicin contained within the niosomes. A standard calibration curve was established for doxorubicin concentrations ranging from 1.0 to 50 μM in PBS. The %EE was calculated from the equation *y* = 2.9887*x* + 0.0118, with an *R* ^2^ value of 0.9981.

### 3.2 Cellular uptake

**FIGURE 7:**
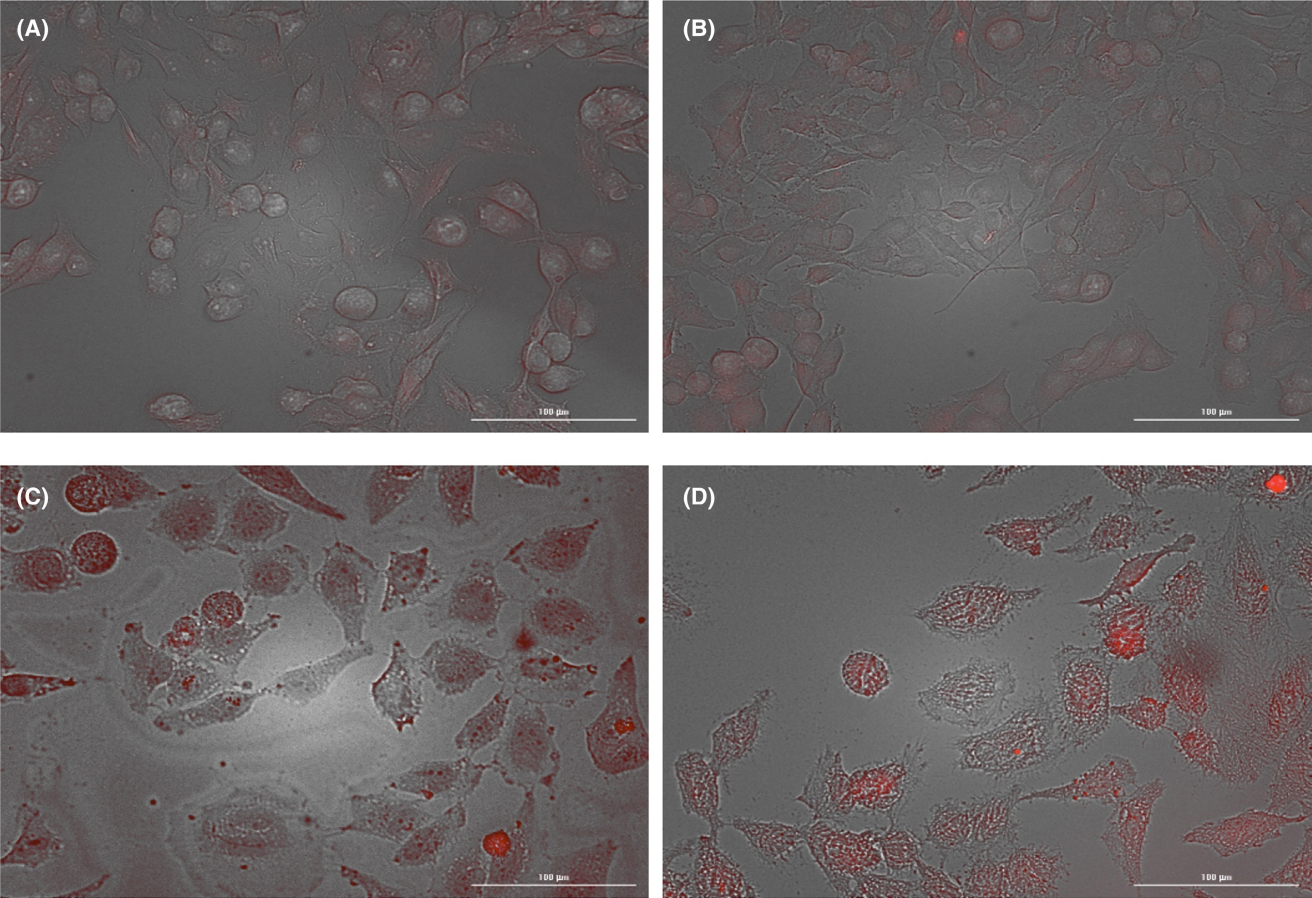
Fluorescent image of Nio/Dox‐Cis/MUC1 on U87 cell line (negative control) (A, B) and HeLa cell line (positive control) (C, D).

Fluorescence microscopy, augmented by flow cytometry, was employed to investigate the interaction of drug‐loaded niosomal vesicles with cells. Recently, fluorescence microscopy has become increasingly pivotal in cellular imaging research,^44^, ^45^ and flow cytometry has established itself as one of the most accurate and widely used techniques for studying cellular uptake.^46^, ^47^ The robust fluorescence properties of doxorubicin, which emits at 620 nm, have facilitated effective imaging studies.^48^ Niosomes enhance cellular penetration, while MUC‐1 aptamers increase the affinity toward HeLa cells.^49^ As indicated in Figure 7, MUC‐1‐positive HeLa cells internalized a greater number of vesicles compared with MUC‐1 negative U87 cells, confirming the efficacy of active targeting mechanisms.

Doxorubicin tends to accumulate significantly within the cell nucleus and can be easily identified using a red fluorescent filter.^50^ In fluorescent images of Nio/Dox‐Cis/MUC1, the vesicles are predominantly observed in the nuclear region of the cells. These observations confirm that doxorubicin maintains its functional integrity post‐conjugation, suggesting its potential role as a nucleus‐specific targeting agent.

### 3.3 Cytotoxicity

**FIGURE 8:**
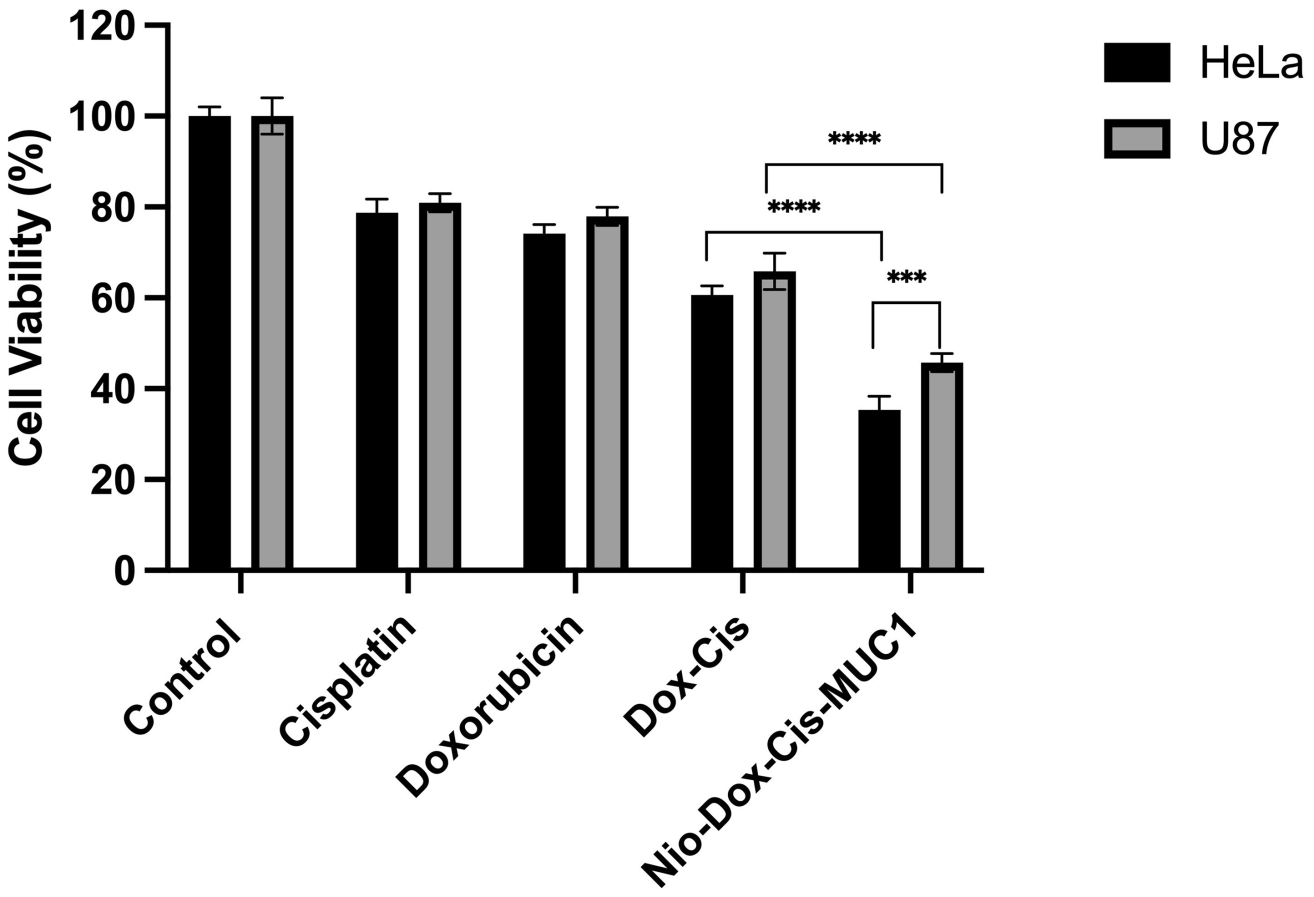
Toxic effect of Nio/Dox‐Cis/MUC1 on HeLa and U87 cell lines (***p < 0.001, ****p < 0.0001).

Recent years have seen a surge in research focusing on combination drug therapies and combination treatments. Researchers are increasingly utilizing the simultaneous effects of two distinct cancer drugs or therapies to overcome cancer's formidable ability to develop resistance to treatments, leading to a growing number of publications on this subject annually. In this context, cytotoxicity analyses were conducted to measure the combined effects of conjugated forms of cisplatin and doxorubicin. Based on the calculations, a dose of 2.5 μM was found to be effective for Nio/Dox‐Cis/MUC‐1 conjugate, and the same doses were used for the conjugate and drug individuals. The results indicated that the conjugated drug formulation was more effective compared with the individual drugs. Specifically, for the HeLa cell line, the drugs and conjugated formulation affected cell viability at levels of 59.878 ± 2.144 and 38.503 ± 1.407, and for the U87 cell line, at levels of 65.943 ± 4.809 and 46.653 ± 1.297, respectively. Additionally, niosomal cargoes targeted with MUC‐1 aptamer significantly reduced cell viability in the HeLa cell line compared with the U87 cell line (Figure 8). This increased cytotoxic effect can be attributed to the higher affinity of MUC‐1 aptamers for HeLa cells relative to U87 cells,^51^ and the enhanced cellular uptake of encapsulated nanoparticles. Moreover, the conjugation of the drugs likely led to a higher accumulation within the cell nucleus, enabling doxorubicin to act as a targeting agent that facilitates the transport of cisplatin directly to the nucleus, thereby exerting a synergistic effect on the targeted cells. In a similar study, camptothecin and chlorambucil were conjugated together by a disulfide linkage, and the resulting new drug molecule was shown to overcome multi‐drug resistance and be more effective compared with the drugs used individually.^52^ In another study, methotrexate and capecitabine were conjugated, and the results were evaluated through in silico calculations, leading to the conclusion that this conjugate could be an effective drug molecule for breast cancer treatment.^53^

## 4 CONCLUSION

In this study, a Dox‐Cis conjugate was created as an alternative treatment method, and biocompatibility was subsequently increased using niosomes targeting a specific cell line with MUC‐1 aptamers. Accurate characterization of the Dox‐Cis conjugates using FTIR and LC‐Q‐TOF/MS confirmed the conjugation process. Additionally, rigorous evaluation of the niosomes for size, zeta potential, and homogeneity confirmed their suitability for effective intracellular delivery. Intracellular uptake experiments confirmed the hypothesis that Nio/Dox‐Cis/MUC‐1 niosomes exhibit enhanced internalization by cancer cells specifically expressing MUC‐1, thereby validating the targeted approach. This result also correlates with cytotoxicity results. These findings provide new perspectives on drug delivery systems, paving the way for the development of more effective niosomal formulations in cancer treatment. Importantly, the study suggests that this system has the potential to overcome drug resistance, a major challenge in chemotherapy, by enabling targeted and sustained delivery of conjugated drugs to cancer cells. However, this study has certain limitations, including the need for further in vivo validation and investigation of long‐term effects. Future research should focus on overcoming these limitations and optimizing the carrier system to further improve its performance. By providing a robust platform for targeted drug delivery, this research supports the advancement of personalized medicine and offers new avenues for improving patient outcomes in cancer treatment.

## AUTHOR CONTRIBUTIONS

**Firat Baris Barlas:** Conceptualization (equal); data curation (equal); formal analysis (equal); investigation (equal); methodology (equal); project administration (equal); resources (equal); software (equal); supervision (equal); validation (equal); visualization (equal); writing – original draft (equal); writing – review and editing (equal). **Bilge Olceroglu:** Data curation (equal); methodology (equal); software (equal); validation (equal); writing – original draft (equal); writing – review and editing (equal). **Didem Ag Seleci:** Methodology (equal); project administration (equal); software (equal); supervision (equal); validation (equal); visualization (equal); writing – original draft (equal); writing – review and editing (equal). **Zinar Pinar Gumus:** Formal analysis (equal); methodology (equal); software (equal); validation (equal); visualization (equal); writing – original draft (equal); writing – review and editing (equal). **Pinar Siyah:** Formal analysis (equal); investigation (equal); methodology (equal); software (equal); validation (equal); writing – original draft (equal); writing – review and editing (equal). **Meriam Dabbek:** Data curation (equal); methodology (equal); software (equal); validation (equal); writing – original draft (equal); writing – review and editing (equal). **Georg Garnweitne:** Funding acquisition (equal); investigation (equal); software (equal); supervision (equal); visualization (equal); writing – original draft (equal). **Frank Stahl:** Formal analysis (equal); funding acquisition (equal); methodology (equal); project administration (equal); supervision (equal); visualization (equal); writing – original draft (equal); writing – review and editing (equal). **Thomas Scheper:** Funding acquisition (equal); investigation (equal); methodology (equal); supervision (equal); validation (equal); writing – original draft (equal); writing – review and editing (equal). **Suna Timur:** Funding acquisition (equal); investigation (equal); project administration (equal); supervision (equal); validation (equal); visualization (equal); writing – original draft (equal); writing – review and editing (equal).

## CONFLICT OF INTEREST STATEMENT

The authors declare that there is no conflict of interest regarding the publication of this paper.

## CONSENT

All authors have read and approved the final manuscript. They consent to the publication of this work.

## Data Availability

The data supporting the findings of this study are available within the article. Additional data that support the findings of this study are available from the corresponding author upon reasonable request.
